# Supplementary figures and images for: Rapid Inactivation of SARS-CoV-2 Variants by Continuous and Intermittent Irradiation with a Deep-Ultraviolet Light-Emitting Diode (DUV-LED) Device
Source: Pathogens. 2021 Jun 15;10(6):754. doi: 10.3390/pathogens10060754 (PMC8232135; doi:10.3390/pathogens10060754)

Scheme 1

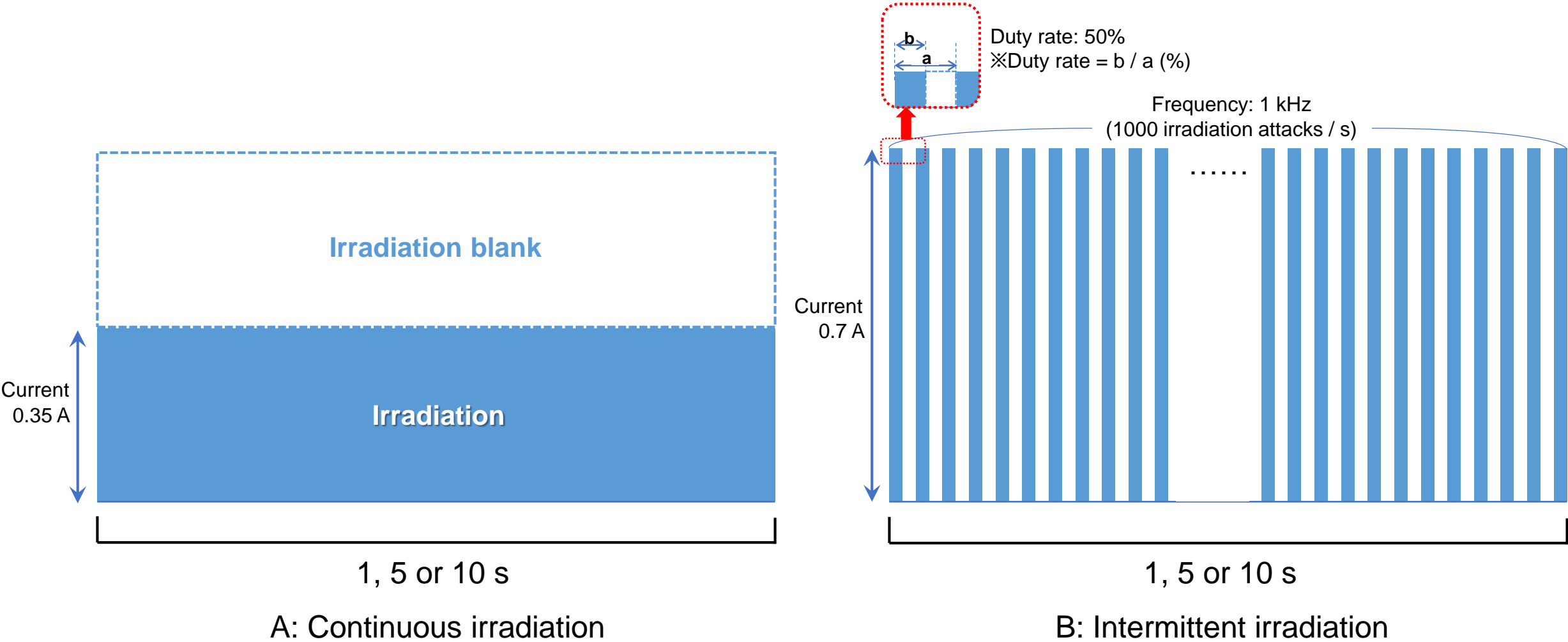

Supplement: Supplementary file 1 [file pathogens-10-00754-s001.zip › pathogens-1234701-supplementary.pdf]
